# Phosphatidylethanol in post‐mortem blood: A comparative study of blood matrices and its stability at routine storage conditions

**DOI:** 10.1111/1556-4029.70106

**Published:** 2025-06-26

**Authors:** Jeremai Hose, Hilke Andresen‐Streichert, Mario Thevis, Markus A. Rothschild, Martin Juebner

**Affiliations:** ^1^ Institute of Legal Medicine Faculty of Medicine and University Hospital, University of Cologne Cologne Germany; ^2^ Centre for Preventive Doping Research/Institute of Biochemistry German Sport University Cologne Cologne Germany

**Keywords:** alcohol biomarker, dried blood spots, femoral vein blood, forensic toxicology, heart blood, phospholipase D

## Abstract

Determination of alcohol markers in post‐mortem cases can be useful to classify drinking habits and potential alcohol habituation prior to death. Phosphatidylethanol (PEth) is a direct alcohol marker and is already commonly used in a variety of contexts. However, its use in the field of post‐mortem toxicology has been scarcely investigated so far. To evaluate its validity, PEth was determined in routinely collected post‐mortem heart blood and femoral vein blood. The stability of PEth under routine storage conditions (−20°C) for a period of 60 days was examined. Post‐mortem blood was collected during medicolegal autopsies and aliquoted. Parts of the samples were used to create dried blood spots (DBS) directly after collection. Further DBS were created using aliquots stored at −20°C on days 1, 2, 7, 14, 30, and 60. LC–MS/MS was used for quantitative PEth analysis, and initial blood alcohol was determined using GC‐FID. Blood was collected from 50 different post‐mortem cases. The heart blood/femoral blood ratio of PEth concentrations varied from 0.32 to 2.36 (mean = approx. 1.00), indicating a good comparability in total but a strong interindividual variation. In all PEth‐positive samples, the PEth concentrations increased by approximately 20% after 24 hours and 70% after 60 days. Post‐sampling formation of PEth was also found in blood samples without detectable amounts of ethanol. Neither storage at −80°C nor the addition of sodium metavanadate led to satisfactory stability of PEth. Based on our findings, the (sole) use of PEth for post‐mortem toxicology caseworks is not recommended.

**Highlights**

- PEth values in post‐mortem heart and femoral vein blood show high interindividual variability.
- PEth concentrations in post‐mortem blood increase when samples are stored at −20°C.
- The PEth increase appears to be independent of the presence of ethanol in blood samples.
- PEth values in post‐mortem blood samples most likely do not represent ante‐mortem concentrations.

## 1 INTRODUCTION

Ethanol is a centrally acting and psychoactive substance, which can negatively affect and impair cognitive as well as motor functions. As it represents the most commonly used drug worldwide, it is often involved in the field of forensic toxicology, including post‐mortem caseworks, as it can considerably contribute to or even cause death [1, 2, 3]. Due to its volatile properties and rapid metabolism, however, ethanol is detectable in blood for short periods only [4]. This can be an issue in cases with longer time intervals between alcohol intake and death. Ethanol can also be formed post‐mortem due to decomposition processes of the human body [5], therefore further complicating the interpretation of blood alcohol concentrations (BAC) in these cases.

Direct alcohol biomarkers, such as ethyl glucuronide (EtG), can be advantageous compared to the sole BAC determination, especially due to their longer detectability after alcohol consumption. These biomarkers can provide useful insights into an individual's drinking behavior and possible alcohol habituation. In the field of post‐mortem toxicology, they might even help to differentiate ante‐mortem alcohol consumption and post‐mortem formation of ethanol [6, 7, 8, 9].

Phosphatidylethanol (PEth) has proven useful to verify alcohol intake. It is routinely used for living individuals to assess alcohol abstinence or drinking behavior in the fields of, for example, driving fitness, child custody, liver transplantation or addiction medicine [10]. PEth is a group of phospholipids formed in the lipid membranes of erythrocytes by the enzyme phospholipase D (PLD). Under physiological conditions, the PLD catalyzes the hydrolysis of phosphatidylcholine to produce phosphatidic acid and choline [11]. However, if ethanol is present, it reacts as a co‐substrate instead of water due to its higher affinity to the PLD [12], resulting in the formation of PEth [13, 14]. There are currently 48 known homologues of PEth [15], including PEth 16:0/18:1 being the most abundant and therefore the one routinely used for assessment of alcohol consumption. Other homologues, such as PEth 16:0/18:2, can support the interpretation and validity of PEth 16:0/18:1 results [16, 17].

Stability of PEth in whole blood at room temperature [18] and at −20°C [19, 20] is limited and depicts one of the main challenges in routine analysis. For longer storage periods, whole blood samples should be stored at −80°C [20, 21]. However, PEth has been shown to be stable in dried blood spots (DBS) for several weeks or months when stored at room temperature [20, 22, 23].

Although PEth is commonly used in a variety of contexts, only very few data exist for its applicability and stability in post‐mortem cases. Hansson et al. [24] concluded that PEth in post‐mortem blood can be used as an indication of previous alcohol abuse; however, having not taken into account any time‐ and storage‐dependent concentration changes. The objective of our study was to evaluate the applicability and validity of PEth in routinely collected post‐mortem heart blood (HB) and femoral vein blood (FB) specimens. The comparability of these blood samples, as well as an insight into the ratios of PEth homologues, was pursued. In addition, the stability of PEth in post‐mortem HB and FB samples under routine storage conditions (−20°C) was assessed over a time period of 60 days.

## 2 MATERIALS AND METHODS

### 2.1 Chemicals and reagents

Whatman™ filter papers (903 Protein Saver Cards) from Cytiva (Marlborough, USA) were used for dried blood spots (DBS). PEth 16:0/18:1 (1.0 mg/mL, as free phosphate in methanol), PEth 16:0/18:2 (1.0 mg/mL, as free phosphate in methanol), and PEth 16:0/18:1 – D5 (100 μg/mL, as free phosphate in methanol) were purchased from Cerilliant (Round Rock, USA). Methanol and propan‐2‐ol (both Rotisolv® ≥99.95%, LC–MS Grade) were purchased from Carl Roth (Karlsruhe, Germany). N‐hexane (LiChrosolv®, hypergrade for LC–MS), 2‐methylpropan‐2‐ol (Emsure® ACS), and sodium metavanadate (NaVO_3_; anhydrous) were purchased from Merck (Darmstadt, Germany). Water (HiPerSolv Chromanorm, LC–MS‐Grade) was purchased from VWR (Radnor, USA) and formic acid (p.a. ≥ 98%) from Sigma‐Aldrich (St. Louis, USA).

Stock solutions of both PEth homologues were prepared at concentrations of 2 μg/mL, 20 μg/mL, and 100 μg/mL by dilution with methanol. The internal standard PEth 16:0/18:1–D5 was diluted with methanol to yield a working solution of 50 ng/mL. Reference material and working solutions were stored at −80°C.

### 2.2 Autopsies and blood sampling

**FIGURE 1:**
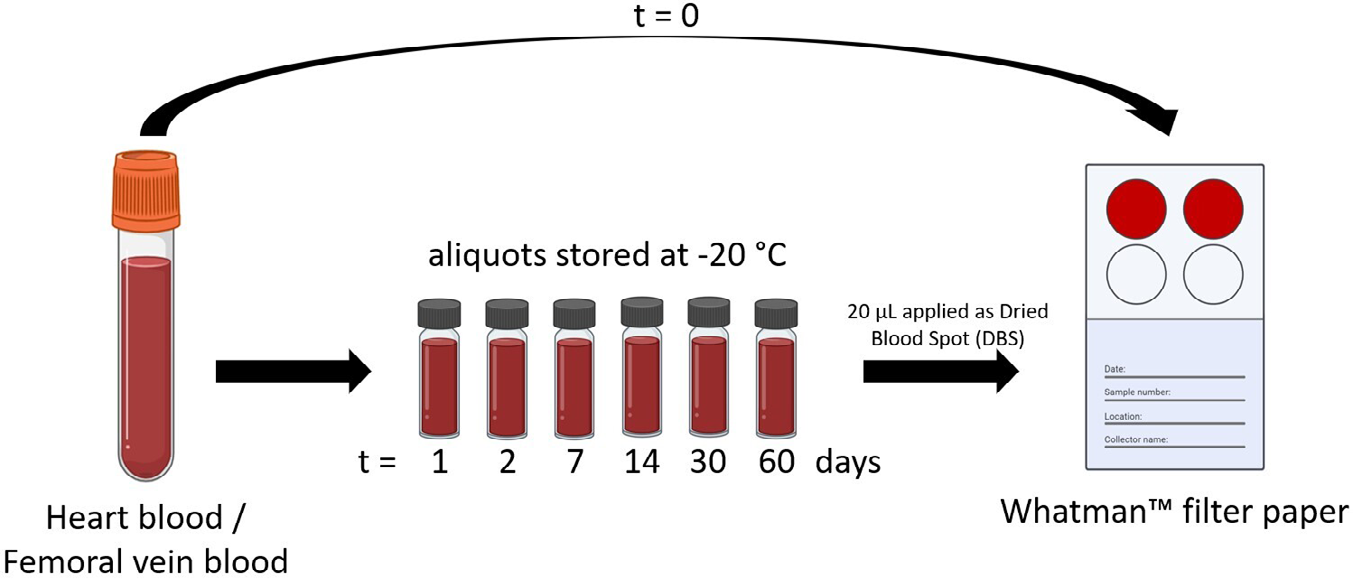
Simplified depiction of sampling handling. Heart blood and femoral vein blood samples were collected during medicolegal autopsies, and 20 μL were applied to Whatman™ filter paper to create dried blood spots (DBS) for day 0 (*t* = 0). Remaining blood was aliquoted in glass tubes and stored at −20°C. After days (*t* =) 1, 2, 7, 14, 30, and 60, the blood aliquots were used to create DBS. All DBS were created in duplicate and stored at room temperature in a zip‐lock bag containing desiccant until analysis using LC–MS/MS.

Medicolegal autopsies were performed according to the guideline of the German Society of Legal Medicine [25] after commission by the public prosecutor's office. HB was obtained by opening the pericardium, removing the pericardial fluid, and then puncturing the atrium using a syringe. FB was taken by cutting the external iliac vein proximal to the inguinal ligament and collecting the blood from the distal cut end. HB and FB were filled in polystyrene tubes and stored at 4°C after autopsy (1 – max. 7 days). Blood was taken in each case and independent of supposed initial blood BAC. After the blood was obtained, DBS on Whatman™ filter paper were created in duplicate using 20 μL each (day 0). Six aliquots of 60 μL each were then separated for all blood samples and stored at −20°C. The temperature of the freezer was controlled daily using a calibrated thermometer. Additional DBS of 20 μL each were created in duplicate from the 60 μL aliquots on days 1, 2, 7, 14, 30, and 60 (Figure 1). After drying for at least two hours, all DBS were stored at room temperature in a zip‐lock bag containing desiccant until analysis. Samples from both HB and FB from all time points of one deceased subject were analyzed in one analytical run.

### 2.3 Attempts on enhancing the storage stability

To further examine and possibly enhance the stability of PEth in post‐mortem blood samples, another experiment was conducted. Heart blood samples of six deceased subjects with positive alcohol findings (0.51–6.17 ‰) were stored for 21 days at different storage conditions: (1) without any additives at −20°C; (2) without any additives at −80°C; (3) with the addition of NaVO_3_ as a PLD inhibitor at −20°C. NaVO_3_ was dissolved in water to obtain a concentration of 0.3 M. Of this solution, 2 μL were then added to 3 mL of heart blood samples to obtain a final concentration of 0.2 mM. The same amount of water was added to the samples without NaVO_3_ addition (conditions 1 and 2) for better comparability. Blood samples were then aliquoted in glass tubes and stored at the temperatures described above. DBS for each blood sample were created on Whatman™ filter paper in duplicate on days 0, 7, 14, and 21. DBS were stored until analysis at room temperature in a zip‐lock bag containing desiccant.

### 2.4 Sample preparation and LC–MS/MS analysis

Sample preparation of the DBS and analysis using LC–MS/MS was carried out according to a previously published method [26]. In short, the *in toto* cut‐out DBS were mixed with deuterated PEth 16:0/18:1 as internal standard and a water/propan‐2‐ol mixture followed by a liquid–liquid extraction using n‐hexane. After centrifugation, the supernatant was evaporated to dryness under a nitrogen stream and reconstituted in 100 μL of the mobile phase A. Analysis of PEth 16:0/18:1 and PEth 16:0/18:2 was carried out using LC–MS/MS (Agilent 1200 Infinity LC® system, using a Acquity UPLC BEH C18 column, coupled to a Sciex QTrap® 6500+ MS) with a limit of detection of 2.5 and 3.2 ng/mL, respectively. The published method was slightly adapted by expanding the calibration curve from 500 ng/mL to 5000 ng/mL and by adding additional quality controls (QCs) at 800 ng/mL and 4500 ng/mL for both homologues. The method was validated, including the additional QCs, according to the guideline of the German Society of Toxicology and Forensic Chemistry (GTFCh) [27].

### 2.5 Determination of initial blood alcohol concentrations using GC‐FID

Initial blood alcohol concentrations (BACs) were determined using a gas chromatography—flame ionization detector (GC‐FID) method with a limit of detection of 0.05 ‰. After centrifugation of blood samples, 50 μL of the serum‐like supernatant were added to 20 mL headspace vials along with 500 μL of 2‐methylpropan‐2‐ol [0.04%] as an internal standard. The headspace vials were then airtight crimped and placed on the instrument's sampler. A duplicate measurement was carried out on two different GC instruments (PerkinElmer Gas Chromatograph Clarus® 500, using an Elite‐BAC capillary column (30 m x 0.32 mm x 0.60 μm) and Clarus® 590, using a ZB‐BAC 1 capillary column (30 m x 0.32 mm x 1.8 μm)) each giving four values in total. The mean of these four values was calculated. BACs were determined in FB. In cases where no FB was obtained, the determination was carried out in HB.

### 2.6 Testing for normal distribution and statistical significance

Data obtained in this study were tested for normal distribution using a Kolmogorov–Smirnov test with a significance level of α = 0.05. A Brown‐Forsythe test was used to test equality of group variances. One‐way ANOVA using a significance level of α = 0.05 was performed if normal distribution and equality of group variances were confirmed.

## 3 RESULTS

Samples were collected from 50 deceased subjects, 30 male and 20 female, with an age range of 0.5 to 89 years. For 39 subjects, both blood samples could be obtained. For 7 subjects, only HB could be obtained, and for 4 subjects, only FB.

Initial PEth concentrations above the limit of detection were found in 42 cases, ranging from approx. 3.0 to 2616 ng/mL for PEth 16:0/18:1 and from approx. 3.7 to 1101 ng/mL for PEth 16:0/18:2. No initial BAC but detectable initial PEth concentrations were found in 26 out of these 42 cases. Measurable initial BACs were found in 16 out of the 50 cases, ranging from approx. 0.09 to 2.52 ‰. In all BAC‐positive blood samples (>0.05 ‰), PEth was detectable. In 8 specimens, neither initial ethanol nor initial PEth was found.

### 3.1 Stability of PEth at routine storage conditions (−20°C)

**FIGURE 2:**
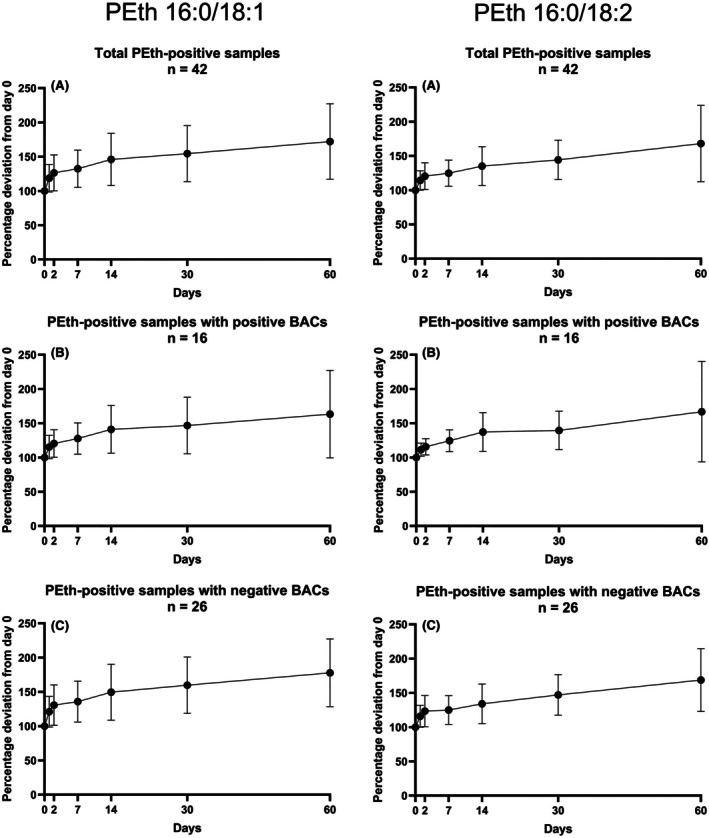
Stability of PEth 16:0/18:1 (left column) and PEth 16:0/18:2 (right column) in post‐mortem heart blood (HB). From top to bottom, the graphs depict the mean values and corresponding variances at each time point. (A) For total initial PEth‐positive samples (including samples with and without detectable blood alcohol concentrations (BACs)). (B) For PEth‐positive samples with positive BACs. (C) For PEth‐positive samples with negative BACs. HB samples were applied as dried blood spots after medicolegal autopsy (day 0), and aliquots were then stored at −20°C. Further DBS were created after 1, 2, 7, 14, 30, and 60 days. All samples belonging to one post‐mortem subject were analyzed in one analytical run. PEth values on day 0 were normalized to 100%, and the percentage deviation for the other sampling days is depicted.

**FIGURE 3:**
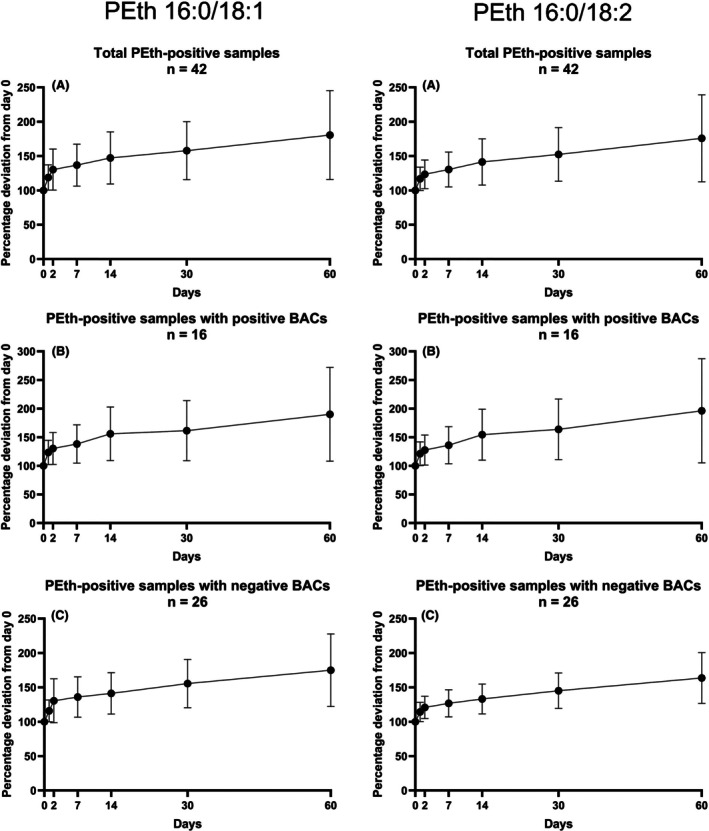
Stability of PEth 16:0/18:1 (left column) and PEth 16:0/18:2 (right column) in post‐mortem femoral vein blood (FB). From top to bottom, the graphs depict the mean values and corresponding variances at each time point. (A) For total initial PEth‐positive samples (including samples with and without detectable blood alcohol concentrations (BACs)). (B) For PEth‐positive samples with positive BACs. (C) For PEth‐positive samples with negative BACs. FB samples were applied as dried blood spots after medicolegal autopsy (day 0), and aliquots were then stored at −20°C. Further DBS were created after 1, 2, 7, 14, 30, and 60 days. All samples belonging to one post‐mortem subject were analyzed in one analytical run. PEth values on day 0 were normalized to 100%, and the percentage deviation for the other sampling days is depicted.

The percentage deviations from day 0 (sampling after autopsy, normalized to 100%) of the determined mean values of PEth concentrations in HB (Figure 2) and in FB (Figure 3) were plotted against the observed time points. Furthermore, in addition to all positive specimens (*n* = 42), samples with detectable amounts of ethanol (positive BACs, *n* = 16) and those without detectable amounts of ethanol (negative BACs, *n* = 26) were displayed separately.

Independent of the initial presence of ethanol in blood, mean PEth concentrations increased by approx. 20% after 24 hours (day 1). After two days, the mean values exhibit a diminished rate of increase, having increased by approx. 50% after 30 days post‐sampling. After 60 days, mean PEth concentrations of both homologues increased by approx. 70% in HB as well as FB. High variances in mean values at each time point were observed across all cases.

In 3 of the 8 specimens, in which no initial PEth was detected, concentrations rose above the limit of detection during the observed time period. Highest measured concentrations in these specimens after 60 days were 16 ng/mL for PEth 16:0/18:1 and approximately 4.7 ng/mL for PEth 16:0/18:2.

### 3.2 Attempts on enhancing the storage stability

**FIGURE 4:**
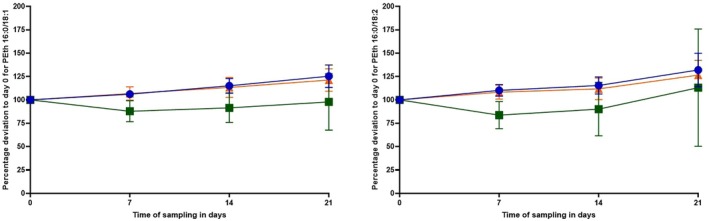
Influence of the storage conditions on the PEth 16:0/18:1 (left) and PEth 16:0/18:2 (right) concentrations (*n* = 6). The graphs depict the mean values and corresponding variances for each time point. Post‐mortem heart blood (HB) samples were applied as dried blood spots after medicolegal autopsy (day 0), and aliquots were then stored at different storage conditions: at −20°C without addition of NaVO_3_ (blue circles), at −80°C without addition of NaVO_3_ (orange triangles) and at −20°C with addition of NaVO_3_ [0.2 mM] (green squares). Further DBS were created after 7, 14, and 21 days. All samples belonging to one post‐mortem subject were analyzed in one analytical run.

Concentrations of both PEth homologues increased in HB samples stored at −20°C and −80°C. After 7 days, an increase of approx. 5–10% of the mean was observed, approx. 10–15% after 14 days and approx. 20–30% after 21 days. The percentage deviations from initially measured PEth values (day 0) in dependence on the time can be found in Figure 4 for both homologues.

By contrast, in blood samples stored at −20°C with the addition of NaVO_3_, PEth concentrations initially decreased. On day 14 (for PEth 16:0/18:1) and on day 7 (for PEth 16:0/18:2), only 85% and 78% of the initial values were detectable, respectively. After that, the concentrations in those samples appeared to increase again.

### 3.3 Comparability of heart blood (HB) and femoral vein blood (FB)

**FIGURE 5:**
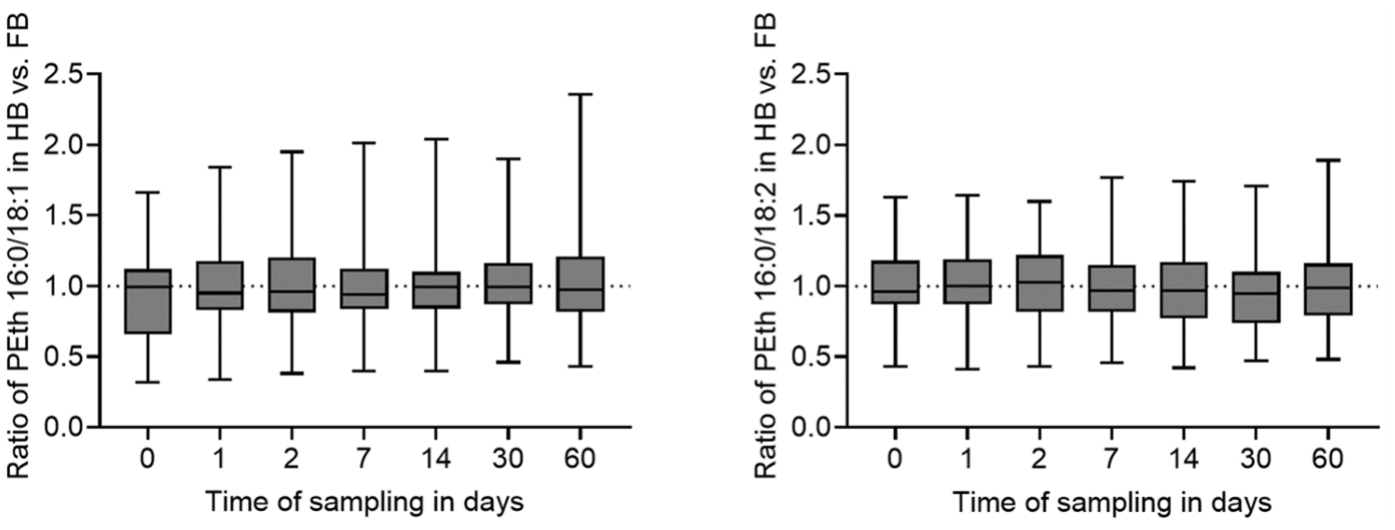
Comparability of heart blood (HB) and femoral vein blood (FB) for the PEth homologues 16:0/18:1 (left) and 16:0/18:2 (right). Concentrations of PEth homologues (*n* = 24) in HB were divided by the concentrations of the corresponding PEth homologue in FB to calculate the HB/FB ratio for each observed time point.

Both HB and FB were obtained in 39 of the 50 cases. Out of these, there were 24 cases that showed PEth values above the limit of quantification in both blood matrices and could therefore be used for comparison. Concentrations of PEth in HB were divided by the concentrations of the corresponding PEth homologue in FB to calculate the ratio, followed by the determination of mean and median values. Data are depicted for each time point in Figure 5 for both PEth 16:0/18:1 and PEth 16:0/18:2.

For PEth 16:0/18:1, the mean values of the HB/FB‐ratio for the time points ranged from 0.95 to 1.03, and the median values ranged from 0.94 to 0.99. Overall, the HB/FB‐ratios for PEth 16:0/18:1 fluctuated between 0.32 (day 0) and 2.36 (day 60). For PEth 16:0/18:2, the mean values of the HB/FB‐ratio for the time points ranged from 0.96 to 1.01, and the median values ranged from 0.95 to 1.03. The lowest and highest observed HB/FB‐ratios for PEth 16:0/18:2 were 0.41 (day 1) and 1.89 (day 60), respectively.

The Kolmogorov–Smirnov test confirmed a normal distribution of data for each homologue at each observed time point. The Brown‐Forsythe test confirmed equality of group variances. The one‐way ANOVA test did not show any significant differences (*p* > 0.05) of HB/FB ratios between the observed days for neither PEth 16:0/18:1 nor PEth 16:0/18:2.

### 3.4 Ratio of PEth 16:0/18:1 and PEth 16:0/18:2 depending on storage time

**FIGURE 6:**
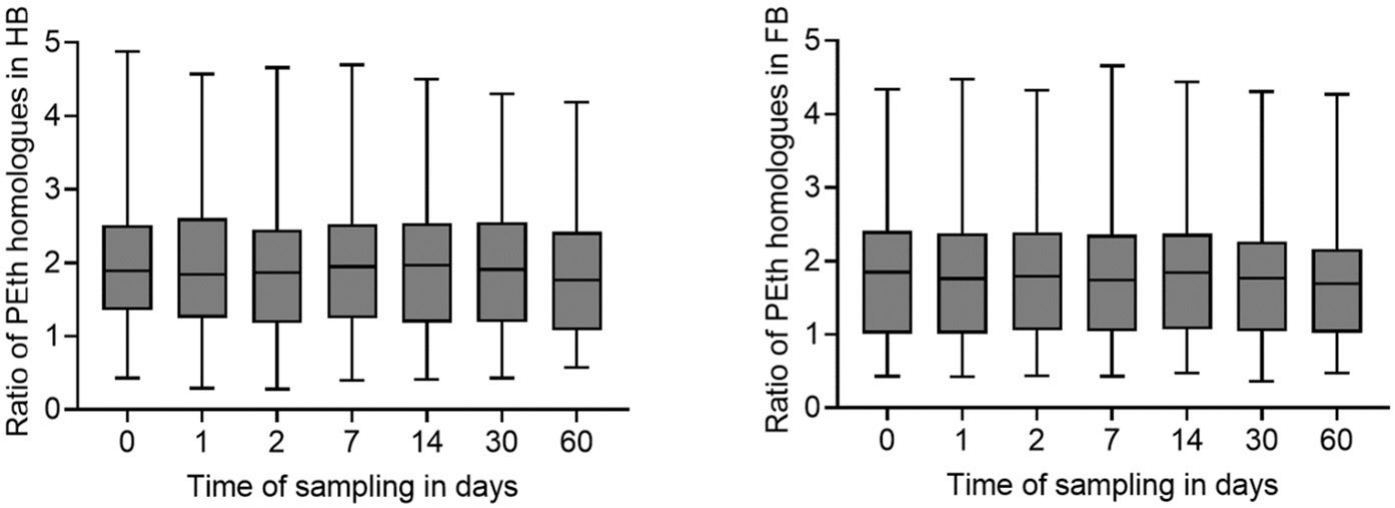
Ratio of PEth 16:0/18:1 vs. PEth 16:0/18:2 in heart blood HB (left, *n* = 27) and femoral vein blood FB (right, *n* = 25). Concentrations of PEth 16:0/18:1 were divided by the concentrations of PEth 16:0/18:2 in each blood matrix and for each observed time point.

HB was obtained in 46, and FB was obtained in 43 of the 50 cases. Out of these, 27 HB samples and 25 FB samples showed values above the limit of quantification for both homologues and could therefore be used for comparison. Concentrations of PEth 16:0/18:1 were divided by the concentrations of PEth 16:0/18:2 to calculate the ratio for each case, followed by determination of mean and median values. Data for each time point are summarized in Figure 6 for both HB and FB.

In HB, the mean for all time points ranged from 1.87 to 2.06, and the median ranged from 1.77 to 1.97. Overall, the ratios in HB fluctuated between 0.28 (day 2) and 4.88 (day 0). In FB, the mean for all time points ranged from 1.72 to 1.85, and the median ranged from 1.69 to 1.85. The lowest and highest observed ratios in FB were 0.36 (day 30) and 4.66 (day 7), respectively.

The Kolmogorov–Smirnov test confirmed a normal distribution of data for each blood matrix and each observed time point. The Brown‐Forsythe test confirmed equality of group variances. The one‐way ANOVA test did not show any significant differences (*p* > 0.05) in PEth ratios between the observed days, neither in HB nor in FB. Additionally, the one‐way ANOVA did not indicate any statistically significant difference (p > 0.05) in PEth ratios between HB and FB within the respective time points.

## 4 DISCUSSION

Validation of the adapted method was successful. Linearity, accuracy and precision were confirmed for the new calibration range and QCs. Increasing the calibration curve from 500 to 5000 ng/mL proved necessary for post‐mortem cases as initial PEth 16:0/18:1 concentrations up to 2616 ng/mL were found. During storage of 60 days, the concentrations increased up to 3951 ng/mL.

Initial detected BAC values ranged from 0.09 to 2.52 ‰. Especially at the lower BAC values, a possible post‐mortem formation of ethanol must be taken into consideration. According to the review by O'Neal et al. [5], post‐mortem ethanol formation can lead to BAC values of up to 0.7 ‰ and even 1.5 ‰. BACs exceeding these values were reported in cases with highly favorable conditions for microbial growth.

### 4.1 Stability of PEth at routine storage conditions (−20°C)

Samples from both HB and FB as well as from all time points of one deceased subject were analyzed in one analytical run to minimize systematic errors (e.g. day‐to‐day variability). Due to the use of aliquots for each time point, all samples were frozen and thawed only once, eliminating the need to account for potential freeze–thaw instability.

PEth concentrations increased dependent on time when post‐mortem blood was stored at routine storage conditions of −20°C. The overall curve progression was mainly independent of blood matrix (HB or FB) and PEth homologue. Post‐sampling formation of PEth in whole blood in the presence of ethanol has already been described in the literature for living individuals [28] as well as deceased subjects [29]. As in this study, the increase of PEth concentrations was nearly the same for samples with (*n* = 16) and without (*n* = 26) detectable BACs, it must be assumed that post‐sampling PEth formation occurs independent of the initial measurable presence of ethanol. Formation of ethanol in blood samples stored at −20°C is not to be expected [30, 31].

Especially in the first few days, PEth values appear to increase more rapidly compared to later storage times, most likely due to a greater availability of phosphatidylcholine, the precursor of PEth. As PEth increased with high interindividual variances by 20% on average after 24 hours already, interpretation of PEth in post‐mortem blood samples should be carried out with great caution.

For this study, it was assumed that PEth remains stable in HB and FB when applied as DBS, as observed for blood of living individuals [20, 22, 23]. However, no studies have been conducted to verify whether post‐mortem blood exhibits the same characteristics. Possible alterations in DBS PEth concentrations during the storage period of 60 days were not considered. At last, it should be noted that any samples analyzed in this study were obtained during routine medicolegal autopsies, without standardization of parameters such as the time interval between death and autopsy. Any potential changes in the concentration of PEth during the post‐mortem interval up to sample collection remain unknown. Hence, the initially measured PEth values presented here do not necessarily represent ante‐mortem concentrations or concentrations at the time point of death. As such standardization is not feasible within routine workflow, this will remain an unknown influencing factor for PEth concentrations in post‐mortem blood samples.

### 4.2 Attempts on enhancing the storage stability

In the samples where no NaVO_3_ was added, the PEth concentrations behaved very similarly regardless of the temperature storage conditions (−20°C or −80°C) and increased to more than 20% of the initial values. This suggests that the PLD enzyme is still active at temperatures of −20°C, which has been described in the literature before [32], but also at temperatures as low as −80°C. When NaVO_3_ was present, there was no detectable initial increase in concentration at −20°C, indicating a successful inhibition of PLD and prevention of post‐sampling PEth formation. Instead, a decrease was observed. This coincides with the frequently reported instability of PEth in the blood of living individuals at this storage temperature in the literature [19, 20]. However, an increase was observed after the initial decrease. Possible reasons could include a reversible inhibition of PLD only and possible ethanol formation in stored post‐mortem blood samples. Additionally, prolonged exposure to cold temperatures such as −20°C could lead to increased hemolysis. As PEth is formed and located in the lipid membranes of erythrocytes [33], this effect might lead to artificially elevated PEth concentrations depending on storage time.

Therefore, the respective approaches of decreasing the temperature or inhibiting the PLD enzyme did not lead to a desired PEth stability. In the future, it should be clarified whether a combined approach (−80°C and addition of NaVO_3_) could lead to satisfactory storage stability.

### 4.3 Comparability of heart blood (HB) and femoral vein blood (FB)

HB and FB are routinely collected during autopsy and commonly analyzed in the field of post‐mortem toxicology. When quantification of drugs or medications is necessary, FB is usually preferred over HB, as it is considered less susceptible to any alterations in the analyte concentration due to post‐mortem redistribution of substances [34].

The mean and median values of HB/FB‐ratios were very close to 1.0 for both homologues and all observed days, indicating a good comparability in total. The consistent ratio over the observed time period supports the previously stated hypothesis that the PEth concentrations in both blood matrices change in a very similar manner over time. Additionally, carried out ANOVA tests indicated that the HB/FB ratios of PEth 16:0/18:1 and PEth 16:0/18:2 in samples stored at −20°C do not change significantly over a time period of 60 days.

The wide detected range of ratios, however, indicates a strong interindividual variation, making the actual comparability of HB and FB or any statements regarding a possible post‐mortem redistribution of PEth inapplicable. PEth concentrations are depending on the hematocrit [35], which was not determined and therefore not accounted for in this study. As the hematocrit in HB and FB might be different, the hematocrit bias depicts an additional limitation when comparing the two blood matrices. Overall, a recommendation of a preferred post‐mortem blood sample for the analysis of PEth cannot be made based on these data.

### 4.4 Ratio of PEth 16:0/18:1 and PEth 16:0/18:2 depending on storage time

The PEth homologues 16:0/18:1 and 16:0/18:2 represent the most and second most abundant homologues formed in human blood after alcohol intake. Therefore, higher PEth 16:0/18:1 values are usually detected compared to PEth 16:0/18:2 when analyzing blood samples [16]. Based on the study by Helander et al. [36], PEth 16:0/18:1 accounts for approx. 37% and PEth 16:0/18:2 for approx. 25% of total PEth, suggesting a ratio of approx. 1.48 in terms of their relative concentrations in blood samples. Dui et al. [37] reported a ratio ranging from 0.3 to 8.8 with an average ratio of 1.4 in blood samples of living individuals. The mean and median values of PEth 16:0/18:1 to PEth 16:0/18:2 ratios observed in this study are above those described in the literature. Similar to Dui et al., a large range of ratios was also observed in this study, indicating strong variations between individuals.

In this study, the ratios of PEth 16:0/18:1 to PEth 16:0/18:2 were calculated from different samples, but the time points of possible alcohol intake, death, and autopsy vary in each case. Additionally, the two PEth homologues have different formation rates and half‐life times [38, 39, 40]. Therefore, it can be assumed that the ratio is dependent on temporal factors, which cannot be influenced and accounted for in this study. Based on our data, it can only be concluded that the ratio of PEth 16:0/18:1 to PEth 16:0/18:2 in HB and FB samples stored at −20°C does not change significantly over a time period of 60 days.

## 5 CONCLUSION

Phosphatidylethanol (PEth) values varied strongly between heart blood and femoral vein blood, considerably limiting their comparability. Concentrations of PEth 16:0/18:1 and PEth 16:0/18:2 increased notably when post‐mortem heart blood and femoral vein blood were stored at −20°C for 60 days. Such an increase could be observed for blood samples containing ethanol, but also for samples without detectable amounts of ethanol. Stability of PEth in post‐mortem blood could not be enhanced by storing samples at −80°C or with the addition of the phospholipase D inhibitor sodium metavanadate (NaVO_3_). Overall, it must be assumed that determined PEth values in post‐mortem blood samples are most likely not representative of initial ante‐mortem concentrations.

In conclusion, the (sole) use of PEth for post‐mortem toxicology casework, for example, for assessment of post‐mortem ethanol formation or classification of drinking habits and potential alcohol habituation, is not recommended. The use of additional markers, such as ethyl glucuronide in urine and hair, should be considered.

## CONFLICT OF INTEREST STATEMENT

The authors have no conflicts to declare.

## ETHICS STATEMENT

Examination of post‐mortem sample material for scientific research purposes has been approved by the public prosecutor's office, Cologne, Germany.

## Data Availability

Data of this study are available from the corresponding author upon reasonable request.
